# Retrospective Analysis of Angiographic Radial Artery Spasm Predictors

**DOI:** 10.3390/life15111759

**Published:** 2025-11-16

**Authors:** Adrian Sebastian Zus, Simina Crișan, Silvia Luca, Daniel Nișulescu, Mihaela-Daniela Valcovici, Oana Pătru, Mihai-Andrei Lazăr, Cristina Văcărescu, Dan Gaiță, Constantin-Tudor Luca

**Affiliations:** 1 Cardiology Department, “Victor Babes” University of Medicine and Pharmacy, 2 Eftimie Murgu Sq., 300041 Timisoara, Romania; adrian.zus@umft.ro (A.S.Z.); silvia.luca0@student.umft.ro (S.L.); mihaela.valcovici@umft.ro (M.-D.V.); oana.patru@umft.ro (O.P.); lazar.mihai@umft.ro (M.-A.L.); cristina.vacarescu@umft.ro (C.V.); dan.gaita@umft.ro (D.G.); constantin.luca@umft.ro (C.-T.L.); 2 Research Center of the Institute of Cardiovascular Diseases Timisoara, 13A Gheorghe Adam Street, 300310 Timisoara, Romania; daniel.nisulescu@umft.ro; 3 Department of Histology, Faculty of Medicine, Vasile Goldis Western University of Arad, 310025 Arad, Romania

**Keywords:** radial artery spasm, vascular access, spasm predictors, radial diameter, pain score

## Abstract

Background: Radial artery spasm remains a frequent complication during transradial coronary and peripheral angiography. Despite its impact on procedural success and patient discomfort, consistent predictors remain elusive, as does a standard definition. Objectives: This study aimed to identify independent clinical, hemodynamic, and anatomical predictors of radial artery spasm using data from a single-operator, real-world cohort of patients undergoing both elective and emergency procedures, utilizing an angiographic definition of radial artery spasm. Methods: A retrospective observational analysis was conducted on 96 patients with successful radial artery access. Radial artery spasm was objectively defined as >50% luminal narrowing on radial angiography. Patient demographics, procedural characteristics, comorbidities, and arterial parameters were analyzed. Univariate and multivariate logistic regression models were used to identify significant predictors. Results: Radial artery spasm occurred in 62.5% of patients. Univariate analysis identified lower height, weight, smaller radial artery diameter, higher pain scores, and lower diastolic blood pressure as associated with radial artery spasm. In multivariate analysis, only lower body weight (β = −0.043, *p* = 0.0307) and smaller radial artery diameter (β = −1.352, *p* = 0.0200) remained independent predictors. Age, sex, and most comorbidities, including diabetes, chronic kidney disease, and peripheral artery disease, showed no significant association. Clinically, these findings suggest that simple pre-procedural assessment of patient weight and radial artery diameter may help operators identify individuals at higher risk of radial spasm, allowing for tailored preventive strategies and potentially improving procedural comfort and success. Conclusions: Our findings suggest that low body weight and small radial artery diameter are significant independent predictors of angiographic radial artery spasm, highlighting the importance of anatomical considerations over demographic or clinical factors. Preprocedural assessment of radial artery size may enhance risk stratification and guide preventive strategies. Further multicenter validation is warranted. Incorporating routine evaluation of radial artery size and body habitus into pre-procedural assessment may help identify patients who could benefit from tailored preventive approaches—such as smaller sheath sizes, increased vasodilator use, or ultrasound-guided puncture—to optimize procedural success and patient comfort.

## 1. Introduction

Radial artery access has become the preferred vascular approach for coronary and peripheral angiography due to its association with lower bleeding risk, earlier ambulation, and improved patient comfort compared to femoral access [1,2]. Despite these advantages, RAS remains a common procedural complication, but data is ununiform because of varying definitions, based on both objective and subjective criteria (operator or angiographic evaluation) [3]. Henceforth, occurrence rates vary to as high as 51% of cases [4]. RAS is a significant cause of patient discomfort, access site failure, procedural delays and further complications [5,6,7,8], and can described as a multifactorial phenomenon, involving patient-specific anatomical and physiological factors, as well as procedural elements such as sheath size and operator technique [9].

Several studies have attempted to identify risk factors for RAS [10,11,12], going as far as recommending risk scores to anticipate high probability of RAS [13,14], yet consistent and generally accepted and recognized predictors remain elusive, particularly in real-world clinical settings. While smaller radial artery diameter, female sex, and pain during cannulation have been variably implicated, the relative contribution of anthropometric parameters, comorbidities, and hemodynamic status has not been conclusively established. Thus, our research further evaluates the association between patient baseline physiological and anatomical characteristics and the occurrence of RAS, using both univariate and multivariate analyses, to help better plan future arterial access strategies and determine prophylactic measures against RAS.

The primary objective of this study was to identify clinical, hemodynamic, and anatomical predictors of RAS in patients undergoing both emergency and elective coronary or peripheral angiography via radial access. We aimed to assess the relationship between RAS and variables including patient demographics (age, sex, height, weight), hemodynamics (blood pressure, heart rate), arterial characteristics (radial artery diameter, pulse grade), and comorbidities (diabetes, chronic kidney disease, peripheral artery disease, dyslipidemia, smoking status). A secondary objective was to determine whether any of these factors remained statistically significant independent predictors of RAS in a multivariate logistic regression model.

Considering that most studies utilize clinical RAS, which is highly subjective, as it is based on operator evaluation of difficulty to maneuver catheters, we opted for angiographic determination of RAS as an objective measurement.

## 2. Materials and Methods

Study design was based on the STROBE checklist for reports of observational studies. All consecutive patients undergoing radial artery access for coronary or peripheral artery angiography or angioplasty at our center, done by the same experienced radial operator, during a period of three months, were included, for a total of 100 cases, undergoing both emergency and planned procedures, in a tertiary referral center for western Romania. Exclusion criteria were the prior failure of radial access site, procedural need for a different arterial access, absence or radial pulse, other patient physical characteristics that precluded radial puncture, and failure to obtain patients consent for the use of their medical information for the scope of research. Thus, our cohort reflects real-world patients, which were enrolled during a three-month period (April–June 2023), having at that time been prospectively selected for a study on the efficacy of topical nitroglycerine in prevention of radial artery spasm (with randomization to topical nitroglycerine and placebo arms based on a random number generator). Out of the original 100 patients, there were 4 cases in which radial puncture and access were not possible, leaving a total of 96 patients. Classic radial artery puncture site (most palpable point) was used, with no distant radial access. No ultrasound guidance was used, and local anesthesia was obtained using 2 mL of 1% lidocaine solution injected subcutaneously at the puncture site, with an additional dose if patient felt increased discomfort after sheath placement. The cut-off value of 50% radial lumen reduction was chosen based on the same value being the most often used cut-off for relevant stenosis in other vascular territories (coronary, peripheral), and as a mean of the values used by other authors in their studies (25% [15] to 75% [16] stenosis). In order to prevent contrast-induced spasm, a diluted solution of contrast (1:1 with normal saline) was gently injected to obtain the angiogram, in an anteroposterior projection. After the procedure was completed, the operator used the built-in software of the Siemens imaging machine, selecting the angiogram in which the whole radial artery was best visible, with calibration based on sheath size. The entire length of the radial artery, from sheath tip to brachial artery, was assessed and degree of stenosis was automatically calculated by the software, by comparing the largest artery diameter (in non-spastic areas) to the narrowest (the area of most severe spasm). As calculations were automatic and machine-based, utilizing the frame that best showed the filling of the lumen of the entire radial artery length, no blinding was used, and a second reader was deemed unnecessary.

In addition to the topical agent that was tested, the operator was free to use any antispastic measures deemed necessary, including intraarterial nitroglycerine or switch to smaller catheters at any time during the procedure, including after sheath placement, in case blood flow was absent or sheath could not be properly advanced, and in response to increased pain felt by the patient. We note that use of routine nitroglycerine intraarterial administration is not practiced in our lab. As the aim of our study is to assess real-world RAS that occurs despite measures used to prevent and treat it (topical or intraarterial nitroglycerine, smaller sheath size, anxiety reducing measures), we have not considered antispastics as confounders, as operators are free to use any measures they see fit on a case-by-case situation.

Patient characteristics that were deemed good candidates for radial artery spasm predictors were noted: emergency or planned procedure, sex, body size (height, weight, body-mass index), radial artery diameter, as measured on invasive radial angiography (defined as the largest diameter measured along the whole length of the radial artery), sheath-to-artery diameter ratio, blood pressure, heart rate, the presence of diabetes, dyslipidemia, peripheral artery disease, chronic kidney disease (eGFR below 60 mL/min/1.72 m squared body surface), smoking status, and objective parameters represented by preprocedural operator grading of radial pulse (poor, adequate and good) and patient discomfort caused by forearm pain during the procedure, assessed by the operator, using a Visual Analog Scale, at 15 to 30 min intervals, with emphasis on forearm pain and not back or chest pain that may be caused by the underlying pathology.

In order to assess potential selection bias and reflect real world patients, exclusion criteria were based on anatomical characteristics that normally steer the operator towards a different access site. Information bias was mostly averted using numerical objective measurements were possible, including definition of radial artery spasm.

The sample size of 96 patients was determined by the total number of consecutive cases undergoing radial artery cannulation performed by the physician during the study period. No sample size calculation was performed, as the study was designed to retrospectively analyze all available data from routine clinical practice.

Quantitative variables were initially analyzed as continuous values. Summary statistics (mean ± standard deviation or median with interquartile range, as appropriate) were calculated, and distributions were visually assessed for normality. For inferential analysis, continuous variables were included in univariate comparisons using Student’s *t*-test or Mann–Whitney U test depending on normality.

For multivariate logistic regression modeling, continuous predictors were retained in their original form to preserve statistical power and avoid loss of information. In particular, variables such as weight, height, radial artery diameter, and self-reported pain score were modeled as continuous to capture linear associations with the probability of radial artery spasm. All statistical analyses were performed using IBM SPSS Statistics version 20.0 software for Windows with a significant *p* < 0.05.

Descriptive statistics were used to summarize the cohort characteristics. Continuous variables were described as mean ± standard deviation or median and interquartile range, depending on distribution. Categorical variables were reported as counts and percentages.

Comparisons between patients with and without radial artery spasm were conducted using the independent samples *t*-test for normally distributed continuous variables and the Mann–Whitney U test for non-normal distributions. Categorical variables were compared using the Chi-square test or Fisher’s exact test, as appropriate.

To identify independent predictors of radial artery spasm and control for confounding, a multivariate logistic regression model was constructed. Variables with a *p*-value < 0.10 in univariate analyses were entered into the multivariate model. Collinearity between variables was assessed before inclusion, and sheath-to-artery ratios was excluded due to very strong collinearity with radial artery diameter. Because pseudo-R^2^ in logistic regression is not directly comparable to linear R^2^, we additionally report discrimination (AUC) and overall accuracy (Brier score), and we assess calibration (intercept and slope). To quantify potential overfitting, we conducted bootstrap internal validation (B = 1000 resamples): in each resample the model was re-fitted and evaluated in the out-of-bootstrap sample; the mean difference between ap-parent and test performance estimated optimism, which we subtracted from the apparent AUC/Brier to obtain optimism corrected metrics. The bootstrap calibration slope was used as a global shrinkage factor to adjust coefficients; the intercept was re-estimated to preserve calibration in the large.

## 3. Results

**Figure 1:**
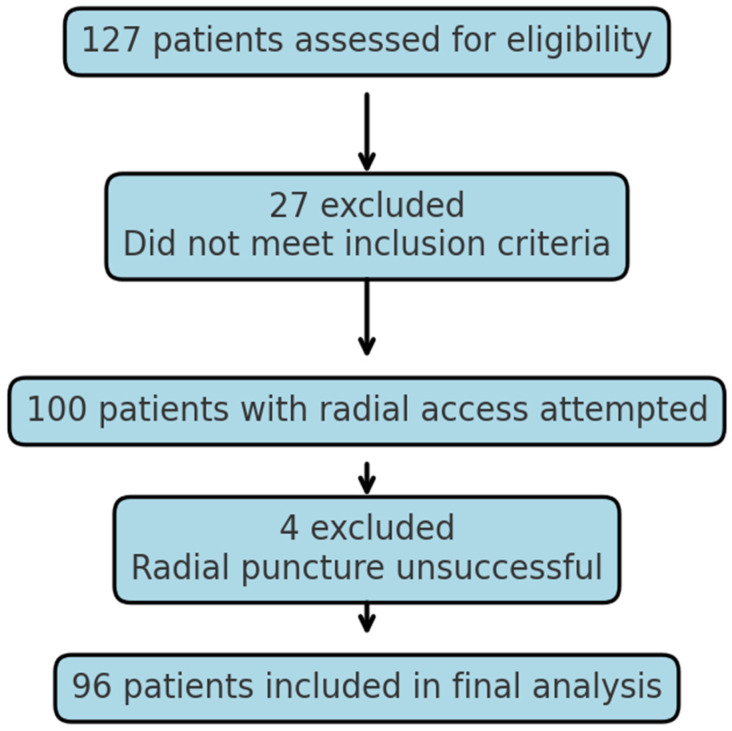
Patient enrollment flow-chart.

A total of 127 cases were performed by the operator during the time period, 27 of which did not meet the inclusion criteria. In total, 100 left or right radial punctures were attempted, and 96 patients had successful radial sheath placement and radial angiography performed (Figure 1).

In total, 60 patients, representing 62.5%, had RAS, and 36 did not (37.5%). The mean age was 63.1 years (±11.3), with a range from 32 to 88 years. Of these, 69.8% were male. The median height was 169.5 cm (IQR: 163–175), and the median body weight was 80 kg (IQR: 71.75–93), with a mean weight of 81.7 kg (±15.2). Exactly half of all patients were acute presenters that had emergency procedures performed, the other half having elective. Dyslipidemia was present in 95.8% of the cohort, DM in 32.3%, PAD in 24%, CKD in 18.8%, and 1 in 3 patients were smokers. Two-thirds of patients had adequate or good radial artery pulse (Table 1 and Table 2).

When comparing patients who developed radial artery spasm (RAS) to those who did not, significant differences were observed in height and reported pain scores. Patients in the RAS group had a lower median height (166 cm; IQR 160–175) compared to those without RAS (171 cm; IQR 165–175.25), *p* = 0.0233. Likewise, the median pain score was significantly higher in the RAS group (4; IQR 3–7) versus the non-RAS group (3; IQR 2–4), *p* = 0.0022. Heart rate did not differ significantly between the two groups (median: 65.5 bpm vs. 72 bpm, *p* = 0.3972) (Table 3).

Age was not significantly different between the two groups (62.9 years mean patient age in RAS group, 63.3 years in no RAS group, *p* = 0.8232), but body weight was markedly lower in RAS patients (77.4 kg vs. 88.9 kg; *p* = 0.0002), and reported pain was higher in RAS group (median 4 with IQR 3–7) compared to those without RAS (median 3 with IQR 2–4), *p* = 0.0022. RAS patients also had significantly smaller radial arteries (mean diameter 2.32 mm vs. 2.67 mm; *p* = 0.0014), and higher sheath-to-artery diameter ratio (*p* = 0.00002). DBP was only marginally different (71 mmHg in RAS vs. 74.9 mmHg in non-RAS; *p* = 0.0429).

No statistically significant differences were found in HR, SBP, sex, age, or most comorbidities such as DM, CKD, PAD, dyslipidemia, or smoking. However, smoking did show a higher absolute frequency in the RAS group (43.3% vs. 25%), suggesting a trend that might reach significance in a larger cohort. Acute presentation with subsequent emergency procedure, as compared to elective, proved not to have any statistical significance as a RAS risk factors (Table 4).

To identify independent predictors of radial artery spasm (RAS), a multivariate logistic regression model was constructed including variables previously significant in univariate analysis: height, weight, radial artery diameter, diastolic blood pressure, and patient-reported pain score (Table 5). Sheath-to-artery diameter ratio was not used because of increased collinearity with radial artery size, owing to the fact that most cases (95%) were performed using 6 French sheaths. The model was statistically significant overall (*p* < 0.0001, pseudo-R^2^ = 0.211). In this analysis, lower body weight (β = −0.043, *p* = 0.0307) and smaller radial artery diameter (β = −1.352, *p* = 0.0200) emerged as independent predictors of RAS. Each kilogram decrease in body weight was associated with increased odds of RAS, as was each millimeter decrease in radial artery diameter. In contrast, height (β = −0.015, *p* = 0.6188), diastolic blood pressure (β = −0.004, *p* = 0.8811), and pain score (β = 0.191, *p* = 0.1272) did not retain statistical significance in the multivariate model.

**Figure 2:**
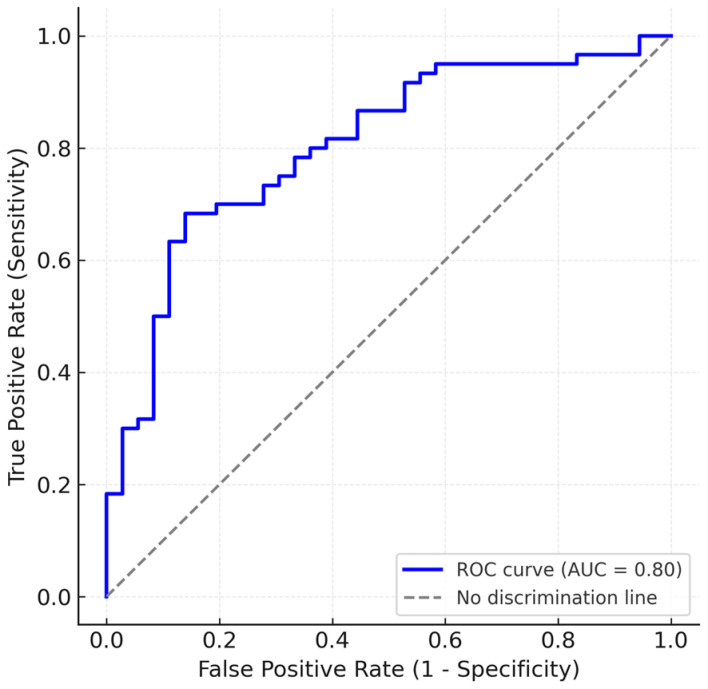
ROC curve for RAS prediction model.

**Figure 3:**
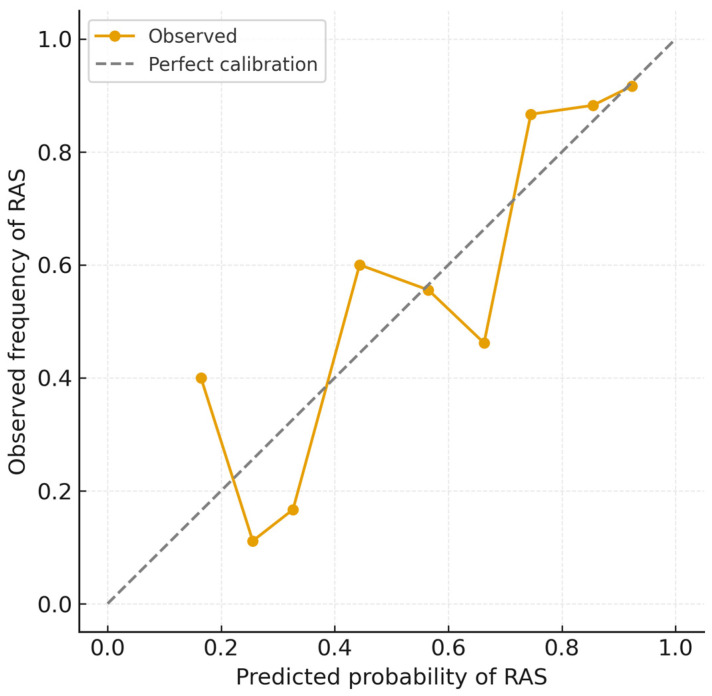
Calibration plot for logistic regression model.

The multivariable logistic regression model demonstrated good discrimination, with an apparent AUC of 0.80 (Figure 2) and a Brier score of 0.17, indicating satisfactory overall predictive accuracy. Bootstrap internal validation (1000 resamples) yielded a mean optimism of 0.04, resulting in an optimism-corrected AUC of 0.77, consistent with moderate and stable discrimination and minimal overfitting. Calibration analysis showed excellent agreement between predicted and observed RAS probabilities (intercept = −0.02, slope = 1.05) (Figure 3), with the calibration plot demonstrating very good alignment along the 45° line. These results confirm that the model’s predictions are both discriminative and well-calibrated for internal validity.

## 4. Discussions

Patients who experienced RAS had a significantly shorter median height (166 cm, IQR 160–175) compared to those without RAS (171 cm, IQR 165–175.25; *p* = 0.0233). This supports the notion that shorter stature may predispose individuals to spasm, likely reflecting a smaller arterial caliber. However, height did not emerge as an independent predictor in multivariate analysis (*p* = 0.6188), suggesting its effect may be mediated through other closely related factors like body weight and radial artery diameter. A predictive score proposed by Giannopoulos et al. [14] indeed uses height < 170 cm as a weighted risk factor, and other studies also state that shorter patients have an increased risk of RAS [17,18].

HR was used as a marker of patient anxiety, stemming from data that suggests anxiety as an inducer of RAS [19], with evidence supporting moderate preprocedural sedation as effective in RAS reduction [18]. Conversely, comparing our cohorts, HR did not differ between patients who developed RAS and those who did not (median 65.5 bpm vs. 72 bpm; *p* = 0.3972). Also related to anxiety and HR is pain, and patients in the RAS group had higher reported pain levels than non-RAS patients (4 with IQR: 3–7, compared to 3 with IQR: 2–4, *p* = 0.0022). This is supported by previous finds [20], but while pain was significantly associated with RAS in univariate analysis of our patient data, it did not remain an independent predictor in multivariate regression (*p* = 0.1272). This may be due to the fact that pain has a bidirectional relationship to RAS: it can both cause it, but it is more likely the consequence of it. Nevertheless, elevated pain scores should alert the operator to the potential presence of RAS and guide real-time management, including administration of intra-arterial vasodilators or downsizing of equipment.

Age was not associated with RAS in our study, a finding that contrasts with some prior assumptions that older age may increase vascular stiffness and procedural difficulty [21]. In our analysis, the mean age of patients in the RAS group (62.9 ± 11.5 years) was not significantly different from that of the non-RAS group (63.3 ± 11.0 years, *p* = 0.8232), suggesting that chronological age alone does not confer increased vulnerability to RAS in a real-world clinical population. This observation aligns with several contemporary studies that have failed to demonstrate a consistent correlation between age and RAS occurrence [11,22].

Patients who experienced RAS had a significantly lower mean weight (77.4 kg) compared to those without spasm (88.9 kg), with a *p*-value of 0.0002. This association remained significant in multivariate analysis (β = −0.043, *p* = 0.0307), suggesting an independent predictive role for weight. These findings are consistent with previous studies that have reported increased incidence of RAS in individuals with lower body mass. Low body surface area has been previously identified as a strong predictor of RAS during transradial procedures [23], and similar conclusions were drawn regarding body mass index [24]. Therefore, patient size—and particularly lower body weight—should be carefully considered when assessing the likelihood of RAS and planning preventive strategies such as vasodilator use, smaller sheath selection, or ultrasound-guided puncture.

SBP did not prove to be associated with RAS, and DBP was only marginally different (71 mmHg in RAS vs. 74.9 mmHg in non-RAS; *p* = 0.0429), which may indicate autonomic vascular tone differences or could simply be a statistical anomaly, which lost its statistical significance after multivariate analysis (*p* = 0.8811).

In our cohorts, acute presentation was not associated with a higher incidence of radial artery spasm. This finding contrasts with the assumption that emergency interventions—often performed under higher stress conditions—might predispose to increased vasospasm, as some data suggests [25]. Similarly, previous studies have noted female sex as a risk factor for RAS [19,26], but this was not the case in our data (*p* = 1). In our patient group females had statistically significantly lower height (median 162 cm IQR: 158–165 cm) and weight (mean: 72 kg ± 13.6) compared to males (172 cm IQR: 168–178 cm, P < 0.0001; 85.6 kg ± 14.5, *p* < 0.0001), such that any sex-based differences are probably confounded by anthropometric differences. In addition, wrist circumference appears to be more tightly related to radial artery size than other physical characteristics [27].

Preprocedural radial pulse grading did not differ significantly between the RAS and non-RAS groups (*p* = 0.8948), and was not associated with spasm risk in univariate analysis. This is in contradiction to a prior study that found empirical pulse assessment as a predictor for RAS [28]. Our sample size may lack sufficient numbers in each pulse grade subgroup to detect a small effect size, or the subjectivity of pulse grading could be the source of the different results.

No statistically significant differences were found in comorbidities such as DM, CKD, PAD, dyslipidemia, or smoking, even though PAD [14], dyslipidemia [25] and DM [29] have all previously been cited as potential RAS risk factors. However, smoking did show a higher absolute frequency in the RAS group (43.3% vs. 25%), suggesting a trend that might reach significance in a larger cohort, as other studies have proven increased RAS in smokers [18,30]. Mechanistically, smoking is known to induce chronic endothelial dysfunction, promote vasoconstriction, and increase sympathetic tone, all of which can contribute to an increased propensity for arterial spasm.

In our analysis, radial artery diameter was a strong and independent predictor of RAS. Patients who experienced RAS had a significantly smaller mean radial diameter (2.32 mm) compared to those without spasm (2.67 mm, *p* = 0.0014). In multivariate logistic regression, radial artery diameter remained a statistically significant predictor (β = −1.352, *p* = 0.020), even after adjusting for other covariates. The validity of our results is backed up by an adjusted AUC of 0.77 and a satisfactory internal predictive value. These findings are consistent with previous reports indicating that smaller radial artery size increases the risk of spasm due to higher sheath-to-artery ratios, increased wall friction, and mechanical irritation [26,31]. Importantly, our study used angiographically measured radial diameter, offering an objective and direct evaluation rather than clinical surrogates such as pulse palpability or anthropometry. Preprocedural radial artery measurement using non-invasive methods (echocardiography) could potentially help screen for patients at high risk of RAS (based on narrow baseline radial artery), such that preventive measures can be taken, including ultrasound-guided puncture or increased success rate [32].

Looking at the high RAS rates (62.5%), our cohort presented, at the higher end of literature reports, we consider this to be a consequence of study design, as most studies utilize clinical indicators of RAS, which are subjective and operator-dependent, and use standard pre-administration of antispastic medication. Other studies that have used angiographic RAS have varyingly utilized cut-offs from 25% [15] to 75% [16] stenosis, encountering RAS in as low as 5% [18] to as high 51% [4] of cases. This emphasizes the heterogenous definitions for RAS and difficulty in pooling studies together for meta-analysis.

In this study, RAS was defined objectively as >50% luminal narrowing on radial angiography. Although we did not prospectively record operator-perceived spasm or formal indices of procedural difficulty, angiographic RAS in our cohort coincided with greater patient-reported forearm pain (median 4 [IQR 3–7] vs. 3 [IQR 2–4]; *p* = 0.0022), supporting its clinical relevance as a correlate of the bedside spasm phenomenon that operators experience as catheter/wire resistance and patient discomfort. In multivariable analysis, however, pain did not remain an independent predictor, whereas smaller radial diameter and lower body weight did, suggesting that anatomic factors are the principal drivers of clinically meaningful spasm, with pain acting as an accompanying—but not causative—signal. These observations align with prior reports that clinical RAS manifests as patient pain and resistance to device advancement and is mitigated by strategies that lower friction and vascular tone [9,18,23,24].

This was a retrospective, single-center analysis of a consecutive three-month series performed by a single experienced radial operator, which constrains external validity and introduces the possibility of selection and measurement biases despite procedural consistency. The cohort size (*n* = 96 with successful radial access) reflected all eligible cases during the study window; no a priori sample size or power calculation was performed, increasing the risk of type II error for associations with small effect sizes and supporting a hypothesis-generating interpretation of our findings. Although RAS was defined objectively as >50% luminal narrowing on radial angiography and quantified with dedicated software, several elements were operator-dependent: device selection (including sheath strategy), the discretionary use of antispasm measures (our lab does not administer routine intra-arterial nitroglycerin), and peri-procedural sedation/analgesia. Patient forearm pain and pulse grade were also assessed by the operator. These choices may influence both the occurrence and the detection of RAS and therefore introduce potential operator bias and confounding. Prospective, multicenter studies with standardized antispasm protocols, predefined operator-reported outcomes, and an a priori sample size are warranted to validate and extend these observations.

In regard to drawbacks of radial artery angiography, an increase in radiation exposure and contrast volume, forearm pain and risk of dissection are possible caveats.

Although anatomical factors demonstrated the strongest association with RAS in our cohort, it is important to note that spasm is a multifactorial process influenced by vascular tone, endothelial function, patient anxiety, inflammatory status, procedural technique, catheter material and lubricity, and pharmacologic agents administered before or during the procedure. In our study, several parameters—including comorbidities, blood pressure, and smoking—showed trends but did not reach statistical significance, likely due to sample size limitations. Future prospective studies should explore the contribution of these physiological and procedural variables, ideally combining angiographic and clinical definitions of RAS to capture its full clinical spectrum.

## 5. Conclusions

In this single-center observational study, we identified small radial artery diameter and low body weight as independent predictors of angiographically diagnosed RAS during transradial procedures. These findings align with previous studies suggesting that anatomical factors play a central role in the development of RAS, likely due to an increased sheath-to-artery ratio. Procedural pain and diastolic blood pressure were also associated with RAS in univariate analysis, though they did not retain statistical significance in multivariate modeling, which may be due to intervariable correlations. No significant differences were observed in age, sex, DM, PAD, or acute presentation, challenging prior findings. Our data suggest that RAS is more strongly associated with individual body habitus and vascular anatomy than with comorbidities or age.

Overall, our findings suggest that preprocedural assessment of body size and of radial artery diameter may improve risk stratification for RAS, potentially guiding access strategy and anti-spasm measures. From a clinical standpoint, incorporating routine evaluation of radial artery size and body habitus into pre-procedural assessment may help identify patients who could benefit from tailored preventive approaches—such as smaller sheath sizes, increased vasodilator use, or ultrasound-guided puncture—to optimize procedural success and patient comfort. However, further studies, ideally prospective and multicenter, are needed to validate these findings and incorporate them into standardized risk models, and additionally to investigate the significance and utility of angiographically diagnosed RAS, as well as specific cut-offs for spasm definition.

## Figures and Tables

**Table 1:** Numerical values for all patients.

All Patients = 96	Median	IQR	
Height (centimeters)	169.5	163–175	
HR (beats per minute)	70	60–80	
Pain score (0–10 scale)	3	2–6	
	**mean**	**SD**	**CI**
Age (years)	63.1	11.3	60.8 to 65.3
Weight (kg)	81.7	15.2	78.6 to 84.8
SBP (mmHg)	158.3	27.3	152.8 to 163
DBP (mmHg)	72.4	10.7	70.3 to 74.6
Radial artery diameter (mm)	2.45	0.49	2.36 to 2.55
Sheath-to-artery diameter ratio	0.85	0.17	0.81 to 0.88

**Table 2:** Categorical values for all patients.

All Patients = 96		
Acute presentation Male sex	48/96 (50%) 67/96 (69.8%)	
PAD	23/96 (24%)	
CKD	18/96 (18.8%)	
Dyslipidemia	92/96 (95.8%)	
DM	31/96 (32.3%)	
Smoking	35/96 (36.5%)	
Pulse grade	1 = poor	32/96 (33.3%)
	2 = adequate	48/96 (50%)
	3 = good	16/96 (16.7%)
RAS	60/96 (62.5%)	

**Table 3:** Cohort numerical values comparison.

All Patients N = 96	RAS (60/96) Median	RAS (60/96) IQR	No RAS (36/96) Median	No RAS (36/96) IQR	*p*		
Height (centimeters)	166	160–175	171	165–175.25	0.0233		
HR (beats per minute)	65.5	60–75	72	60–86.25	0.3972		
Pain score (0–10 scale)	4	3–7	3	2–4	0.0022		
	**RAS (60/96) mean**	**RAS (60/96) SD**	**RAS (60/96) CI**	**No RAS (36/96) mean**	**No RAS (36/96) SD**	**No RAS (36/96) CI**	***p***
Age (years)	62.9	11.5	59.95–65.89	63.3	11	59.6–67	0.8232
Weight (kg)	77.4	14	73.78–81.02	88.9	14.5	84–93.8	0.0002
SBP (mmHg)	157.5	25.6	150.9–164.1	159.6	30.2	149.4–169.8	0.6959
DBP (mmHg)	71	9.7	68.5–73.5	74.9	11.8	70.9–78.9	0.0429
Radial artery diameter (mm)	2.32	0.47	2.2–2.45	2.67	0.44	2.52–2.82	0.0014
Sheath-to-artery diameter ratio	0.89	0.18	0.85–0.95	0.76	0.11	0.73–0.80	0.00002

**Table 4:** Cohort categorical values comparison.

All Patients N = 96	RAS (60 Patients)	No RAS (36 Patients)	*p*
Acute presentation Male sex	27/60 (45%) 42/60 (70%)	21/36 (58.3%) 25/36 (69.4%)	0.2918 1
PAD	11/60 (18.3%)	12/36 (33.3%)	0.1556
CKD	10/60 (16.7%)	8/36 (22.2%)	0.6854
Dyslipidemia	58/60 (96.7%)	34/36 (94.4%)	1
Diabetes mellitus	19/60 (31.7%)	12/36 (33.3%)	1
Smoking	26/60 (43.3%)	9/36 (25%)	0.1123
Pulse grade	1–21/60 (35%)	1–11/36 (30.6%)	0.8948
	2–29 (48.3%)	2–19/36 (52.8%)	
	3–10 (16.7%)	3–6/36 (16.7%)	

**Table 5:** Multivariate analysis of individual RAS predictors.

Variable	β Coefficient	*p*-Value
Weight (kg)	−0.043	0.0307
Radial artery diameter (mm)	−1.352	0.0200
Height (cm)	−0.015	0.6188
DBP (mmHg)	−0.004	0.8811
Pain score (0–10)	0.191	0.1272

## Data Availability

The original contributions presented in this study are included in the article. Further inquiries can be directed to the corresponding author.

## Abbreviations

The following abbreviations are used in this manuscript:

AUC	area under curve
CI	confidence interval.
DBP	diastolic blood pressure.
DM	diabetes mellitus.
HR	heart rate.
IQR	interquartile range.
RAS	radial artery spasm.
ROC	receiver operating characteristics
SBP	systolic blood pressure.
SD	standard deviation.

## References

[1] Chiarito M., Cao D., Nicolas J., Roumeliotis A., Power D., Chandiramani R., Sartori S., Camaj A., Goel R., Claessen B.E. (2021). Radial versus Femoral Access for Coronary Interventions: An Updated Systematic Review and Meta-analysis of Randomized Trials. Catheter. Cardiovasc. Interv..

[2] Neumann F.-J., Sousa-Uva M., Ahlsson A., Alfonso F., Banning A.P., Benedetto U., Byrne R.A., Collet J.-P., Falk V., Head S.J. (2019). 2018 ESC/EACTS Guidelines on Myocardial Revascularization. Eur. Heart J..

[3] Hamon M., Pristipino C., Di Mario C., Nolan J., Ludwig J., Tubaro M., Sabate M., Mauri-Ferré J., Huber K., Niemelä K. (2013). Consensus Document on the Radial Approach in Percutaneous Cardiovascular Interventions: Position Paper by the European Association of Percutaneous Cardiovascular Interventions and Working Groups on Acute Cardiac Care** and Thrombosis of the European Society of Cardiology. EuroIntervention.

[4] Kim S.H., Kim E.J., Cheon W.S., Kim M.-K., Park W.J., Cho G.-Y., Choi Y.J., Rhim C.Y. (2007). Comparative Study of Nicorandil and a Spasmolytic Cocktail in Preventing Radial Artery Spasm During Transradial Coronary Angiography. Int. J. Cardiol..

[5] Ruiz-Salmerón R.J., Mora R., Vélez-Gimón M., Ortiz J., Fernández C., Vidal B., Masotti M., Betriu A. (2005). Radial artery spasm in transradial cardiac catheterization. Assessment of factors related to its occurrence, and of its consequences during follow-up. Rev. Esp. Cardiol..

[6] Dang D., Dowling C., Zaman S., Cameron J., Kuhn L. (2023). Predictors of Radial to Femoral Artery Crossover During Primary Percutaneous Coronary Intervention in ST-Elevation Myocardial Infarction: A Systematic Review and Meta-Analysis. Aust. Crit. Care.

[7] Da Silva R.L., De Andrade P.B., Dangas G., Joaquim R.M., Da Silva T.R.W., Vieira R.G., Pereira V.C., Sousa A.G.M., Feres F., Costa J.R. (2022). Randomized Clinical Trial on Prevention of Radial Occlusion After Transradial Access Using Nitroglycerin. JACC Cardiovasc. Interv..

[8] Sandoval Y., Bell M.R., Gulati R. (2019). Transradial Artery Access Complications. Circ. Cardiovasc. Interv..

[9] Shroff A.R., Gulati R., Drachman D.E., Feldman D.N., Gilchrist I.C., Kaul P., Lata K., Pancholy S.B., Panetta C.J., Seto A.H. (2020). SCAI Expert Consensus Statement Update on Best Practices for Transradial Angiography and Intervention. Catheter. Cardiovasc. Interv..

[10] Roczniak J., Tarnawski A., Dziewierz A., Glanowski S., Pawlik A., Sabatowski K., Januszek R., Rzeszutko Ł., Surdacki A., Bartuś S. (2024). Radial artery spasms—Angiographic morphology, risk factors and management. Adv. Interv. Cardiol..

[11] Curtis E., Fernandez R., Khoo J., Weaver J., Lee A., Halcomb E. (2023). Clinical Predictors and Management for Radial Artery Spasm: An Australian Cross-Sectional Study. BMC Cardiovasc. Disord..

[12] Meng S., Guo Q., Tong G., Shen Y., Tong X., Gu J., Li X. (2023). Development and Validation of a Nomogram for Predicting Radial Artery Spasm During Coronary Angiography. Angiology.

[13] Gragnano F., Jolly S.S., Mehta S.R., Branca M., Van Klaveren D., Frigoli E., Gargiulo G., Leonardi S., Vranckx P., Di Maio D. (2021). Prediction of Radial Crossover in Acute Coronary Syndromes: Derivation and Validation of the MATRIX Score. EuroIntervention.

[14] Giannopoulos G., Raisakis K., Synetos A., Davlouros P., Hahalis G., Alexopoulos D., Tousoulis D., Lekakis J., Stefanadis C., Cleman M.W. (2015). A Predictive Score of Radial Artery Spasm in Patients Undergoing Transradial Percutaneous Coronary Intervention. Int. J. Cardiol..

[15] Gorgulu S., Norgaz T., Karaahmet T., Dagdelen S. (2013). Incidence and Predictors of Radial Artery Spasm at the Beginning of A Transradial Coronary Procedure. J. Interv. Cardiol..

[16] Numasawa Y., Kawamura A., Kohsaka S., Takahashi M., Endo A., Arai T., Ohno Y., Yuasa S., Maekawa Y., Fukuda K. (2014). Anatomical variations affect radial artery spasm and procedural achievement of transradial cardiac catheterization. Heart Vessel..

[17] Pishgahi M., Mehrabi M.A., Adeli M. (2021). Incidence Rate and Risk Factors of Radial Artery Spasm During Transradial Coronary Angiography. Arch. Men’s Health.

[18] Deftereos S., Giannopoulos G., Raisakis K., Hahalis G., Kaoukis A., Kossyvakis C., Avramides D., Pappas L., Panagopoulou V., Pyrgakis V. (2013). Moderate Procedural Sedation and Opioid Analgesia During Transradial Coronary Interventions to Prevent Spasm. JACC Cardiovasc. Interv..

[19] Ercan S., Unal A., Altunbas G., Kaya H., Davutoglu V., Yuce M., Ozer O. (2014). Anxiety Score as a Risk Factor for Radial Artery Vasospasm During Radial Interventions: A Pilot Study. Angiology.

[20] Da Silva R.L., Moreira D.M., Fattah T., da Conceição R.S., Trombetta A.P., Panata L., Thiago L.E.K.S., Giuliano L.C. (2015). Pain Assessment During Transradial Catheterization Using the Visual Analogue Scale. Rev. Bras. De Cardiol. Invasiva (Engl. Ed.).

[21] Dehghani P., Mohammad A., Bajaj R., Hong T., Suen C.M., Sharieff W., Chisholm R.J., Kutryk M.J.B., Fam N.P., Cheema A.N. (2009). Mechanism and Predictors of Failed Transradial Approach for Percutaneous Coronary Interventions. JACC Cardiovasc. Interv..

[22] Trilla M., Freixa X., Regueiro A., Fernández-Rodriguez D., Brugaletta S., Martin-Yuste V., Jiménez M., Betriu A., Sabaté M., Masotti M. (2015). Impact of Aging on Radial Spasm During Coronary Catheterization. J. Invasive Cardiol..

[23] Bertrand O.F., Rao S.V., Pancholy S., Jolly S.S., Rodés-Cabau J., Larose É., Costerousse O., Hamon M., Mann T. (2010). Transradial Approach for Coronary Angiography and Interventions. JACC Cardiovasc. Interv..

[24] Rathore S., Stables R.H., Pauriah M., Hakeem A., Mills J.D., Palmer N.D., Perry R.A., Morris J.L. (2010). Impact of Length and Hydrophilic Coating of the Introducer Sheath on Radial Artery Spasm During Transradial Coronary Intervention. JACC Cardiovasc. Interv..

[25] Goldsmit A., Kiemeneij F., Gilchrist I.C., Kantor P., Kedev S., Kwan T., Dharma S., Valdivieso L., Wenstemberg B., Patel T. (2014). Radial Artery Spasm Associated with Transradial Cardiovascular Procedures: Results from the RAS Registry. Catheter. Cardiovasc. Interv..

[26] Dahm J.B., Wolpers H.G., Becker J., Hansen C., Felix S.B. (2010). Transradial Access in Percutaneous Coronary Interventions: Technique and Procedure. Herz.

[27] Kotowycz M.A., Johnston K.W., Ivanov J., Asif N., Almoghairi A.M., Choudhury A., Nagy C.D., Sibbald M., Chan W., Seidelin P.H. (2014). Predictors of Radial Artery Size in Patients Undergoing Cardiac Catheterization: Insights from the Good Radial Artery Size Prediction (GRASP) Study. Can. J. Cardiol..

[28] Zencirci E., Değirmencioğlu A. (2017). Can Radial Artery Pulse Grading Predict Radial Artery Spasm During Transradial Approach?. Kardiol. Pol..

[29] Ruiz-Salmerón R.J., Mora R., Masotti M., Betriu A. (2005). Assessment of the Efficacy of Phentolamine to Prevent Radial Artery Spasm During Cardiac Catheterization Procedures: A Randomized Study Comparing Phentolamine vs. Verapamil. Cathet. Cardiovasc. Intervent..

[30] Mercado N., Rubio M., Cisneros M., Trejo S., Giraudo M. (2020). Smoking as an Independent Predictor of Radial Spasm. Rev. Argent. de Cardioangiol. Interv..

[31] Horie K., Tada N., Isawa T., Matsumoto T., Taguri M., Kato S., Honda T., Ootomo T., Inoue N. (2018). A Randomised Comparison of Incidence of Radial Artery Occlusion and Symptomatic Radial Artery Spasm Associated with Elective Transradial Coronary Intervention Using 6.5 Fr SheathLess Eaucath Guiding Catheter vs. 6.0 Fr Glidesheath Slender. EuroIntervention.

[32] Seto A.H., Roberts J.S., Abu-Fadel M.S., Czak S.J., Latif F., Jain S.P., Raza J.A., Mangla A., Panagopoulos G., Patel P.M. (2015). Real-Time Ultrasound Guidance Facilitates Transradial Access. JACC Cardiovasc. Interv..

